# Herbal Medicine (Sihogayonggolmoryeo-Tang or Chai-Hu-Jia-Long-Gu-Mu-Li-Tang) for Treating Hypertension:A Systematic Review and Meta-Analysis

**DOI:** 10.1155/2020/9101864

**Published:** 2020-09-09

**Authors:** Boram Lee, Chan-Young Kwon

**Affiliations:** ^1^Clinical Medicine Division, Korea Institute of Oriental Medicine, 1672 Yuseongdae-ro, Yuseong-gu, Daejeon 34054, Republic of Korea; ^2^Department of Oriental Neuropsychiatry, Dong-eui University College of Korean Medicine, 62 Yangjeong-ro, Busanjin-gu, Busan 47227, Republic of Korea

## Abstract

**Introduction:**

For situations in which effective and safe natural-derived products to treat hypertension are needed, recent studies suggest that an herbal medicine, Sihogayonggolmoryeo-tang (SYM), can improve both hypertension and concurrent mood symptoms. We aimed to evaluate the effectiveness and safety of SYM in treating hypertension.

**Methods:**

Thirteen English, Korean, and Chinese databases were comprehensively searched from their inception to May 2020. Randomized controlled trials (RCTs) using SYM as a monotherapy or adjunctive therapy for hypertension were evaluated. The primary outcome was the systolic and diastolic blood pressure (BP). Descriptive analyses of the relevant data were conducted, and where appropriate data were available, a meta-analysis was performed, and the results were presented as a risk ratio or mean difference with 95% confidence intervals. The risk of bias was assessed using the Cochrane risk of bias tool, and the quality of evidence was evaluated using the Grading of Recommendations, Assessment, Development and Evaluation (GRADE) approach.

**Results:**

Seven RCTs with 711 participants were included. Compared with placebo, SYM significantly lowered systolic and diastolic BP and concurrent depression. SYM significantly lowered systolic and diastolic BP compared with active controls; however, subgroup analysis revealed no differences between SYM and antihypertensives. In addition, SYM significantly decreased the level of concurrent depression compared with antidepressants. There was no consistent difference in BP reduction between SYM combined with antihypertensives and antihypertensives alone. No serious adverse events were reported following SYM administration. Most of the included studies had an unclear risk of bias, and the quality of evidence was generally rated “low.”

**Conclusion:**

Current evidence suggests that SYM may have the potential to lower hypertension and concurrent depressive symptoms without serious adverse events. Additional high-quality, placebo-controlled RCTs should be conducted to confirm the efficacy of SYM.

## 1. Introduction

Hypertension is a major public health challenge worldwide. It is strongly associated with potentially severe conditions including cardiovascular disease and premature death [1]. According to a comprehensive systematic analysis, the proportion of ischemic heart disease disability-adjusted life years attributable to high blood pressure (BP) was 53% in 2010 [2]. In the study, high BP was identified as the leading risk factor that affects the global disease burden, followed by tobacco smoking and alcohol use [2]. The prevalence of hypertension in adults worldwide in 2010 was estimated at 31.1% [3], but the proportion of patients treated and/or managed with proper medication is still low [4]. In addition, about 1 in 5 patients have apparent treatment-resistant hypertension, which is unresponsive to adequate antihypertensives [5]. More than 20% of hypertension patients taking antihypertensives experience side effects such as impotence and emotional distress [6], which are the primary determinants of low adherence to antihypertensives [7]. Decreasing sodium intake, weight loss, increasing physical activity, decreasing alcohol intake, and eating a healthy diet including the Dietary Approaches to Stop Hypertension (DASH) diet are healthy lifestyle strategies to reduce the risk of hypertension [8]. However, the development of hypertension due to poor lifestyle habits such as sedentary behavior is still frequently reported [9]. In other words, hypertension remains a major public health issue, and conventional treatment and management strategies still need to be improved.

Some hypertensive patients are interested in complementary and alternative medicine (CAM) modalities. According to the 2012 National Health Interview Survey and the Adult Alternative Medicine supplement, a combination of hypercholesterolemia, hypertension, diabetes, and obesity as dyads and triads was significantly related to the increased use of CAM such as mind-body interventions, manipulative methods, and energy therapies [10]. It has been reported that some supplements/foods including coenzyme Q10, vitamin D, and polyphenol-rich dark chocolate, and mind-body medicine including qigong, breathing, and meditation may be helpful for reducing BP [11]. Herbal medicine (HM) has been used as a treatment approach in East Asian traditional medicine (EATM) to treat various medical conditions including hypertension-related symptoms for thousands of years in East Asia. Today, HM is receiving much attention as an option to replace or supplement conventional medicines, and its effectiveness has already been demonstrated in some diseases including cardiovascular disease and hypertension [12–14].

Sihogayonggolmoryeo-tang (SYM) is a traditional HM used to treat insomnia, anxiety, irritability, and hypertension-related symptoms. In particular, since this HM is well-known for its mood-stabilizing effects [15–17], it may be helpful in improving not only hypertension but also concurrent mood symptoms. In addition, preclinical evidence suggests that SYM can exert a protective effect on the cardiovascular system by enhancing the function of endothelial progenitor cells, inducing antioxidant effects, and inhibiting vasoconstriction [18–20]. However, the efficacy of SYM in the treatment of hypertensive patients has not been systematically summarized and evaluated. Therefore, we aimed to synthesize the available evidence related to the effectiveness and safety of SYM as a monotherapy or adjunctive therapy for patients with hypertension and to assess the methodological quality of these studies to help clinicians establish evidence-based treatment strategies.

## 2. Materials and Methods

The protocol for this review was registered in the International Prospective Register of Systematic Reviews, PROSPERO (registration number: CRD42020187174). We reported this review in accordance with the Preferred Reporting Items for Systematic Reviews and Meta-Analyses statement [21]. Additionally, we followed the methods of Dr. Lee [22].

### 2.1. Data Sources and Search Strategy

One researcher (BL) comprehensively searched total 13 electronic databases on May 19, 2020: 5 English databases (Medline (via PubMed), EMBASE (via Elsevier), the Cochrane Central Register of Controlled Trials (CENTRAL), the Allied and Complementary Medicine Database (AMED) (via EBSCO), and the Cumulative Index to Nursing and Allied Health Literature (CINAHL) (via EBSCO)), five Korean databases (Oriental Medicine Advanced Searching Integrated System (OASIS), Korean studies Information Service System (KISS), Research Information Service System (RISS), Korean Medical Database (KMbase), and Korea Citation Index (KCI)), and three Chinese databases (China National Knowledge Infrastructure (CNKI), Wanfang data, and VIP). We also searched the reference lists of included studies and Google Scholar to identify additional eligible studies. Grey literature such as degree theses was also included. No language, publication date, and publication status restrictions were imposed. The following search terms were used in PubMed: Hypertension [MH] OR hypertens *∗* [TIAB] OR “blood pressure” [MH] OR “blood pressure” [TIAB] OR blood pressure [TIAB] AND Chai-Hu-Jia-Long-Gu-Mu-Li-Tang [TIAB] OR Chai-Hu-Jia-Long-Gu-Mu-Li-Wan [TIAB] OR Chai-Hu-Jia-Long-Gu-Mu-Li-Pian [TIAB] OR Saiko-ka-ryukotsu-borei-to [TIAB] OR Saiko-ka-ryukotsu-borei-to [TIAB] OR Sihogayonggolmoryeo-tang [TIAB] (Supplement 1).

### 2.2. Inclusion Criteria

#### 2.2.1. Types of Studies

We included only randomized controlled trials (RCTs). We also included RCTs using the expression “randomization” without descriptions of the randomization methods. We excluded RCTs in which a quasirandom method was used for the allocation of treatments such as using alternate allocation or allocation by birth date.

#### 2.2.2. Types of Participants

Studies on hypertension patients, diagnosed according to the international hypertension criteria, were included. There was no restriction on age, sex, race, or comorbidity of participants. We excluded trials that included patients suffering from other serious medical conditions such as cancer, liver disease, or kidney disease.

#### 2.2.3. Types of Interventions

We included studies on SYM as a treatment intervention. HMs are known as “modified HMs” when the components are altered to achieve increased efficacy [23, 24]. Thus, we also included studies in which modified forms of SYM, described as “modified SYM” and containing more than 50% of the components of the original prescription, were used as treatment interventions. Modified SYM in the included studies must contain *Bupleuri Radix*, *Fossilia Ossis Mastodi*, and *Ostreae Testa*, one of the most critical herbal components of SYM. For the dosage form, we considered only oral administration of SYM. Placebo, no treatment, and active controls such as conventional medication were included as control interventions. Studies involving SYM combined with other therapies as treatment interventions were also included if the other therapies were equally used in both the treatment and control groups. However, we excluded studies comparing different types of HM. There was no restriction on the treatment duration of SYM.

#### 2.2.4. Types of Outcome Measures

The primary outcome was the systolic BP (SBP) and diastolic BP (DBP) measured after treatment. The secondary outcomes included (1) symptoms associated with hypertension such as insomnia and anxiety after treatment, (2) total effective rate (TER), calculated secondarily based on improvements in BP or other clinical symptoms, (3) the incidence of adverse events during the study period, and (4) quality of life after treatment.

### 2.3. Study Selection and Data Extraction

We removed duplicates in the search results obtained from the databases and additional sources and screened the titles and abstracts for eligibility using EndNote X8. Afterward, we evaluated the full texts of the eligible articles for final inclusion. For the included studies, we extracted information related to the study characteristics (author, publication year, country, and study design); approval from institutional review boards; informed consent; sample size and the number of dropouts; details about the participants, intervention, and comparisons; duration of the intervention and follow-up; outcome measures; results; and adverse events using a standardized data collection form (Excel 2016, Microsoft, Redmond, WA, USA). We also extracted the components, dosage form, and administration duration and frequency of SYM. The corresponding authors of the included studies were contacted via e-mail if there were insufficient data.

One researcher (BL) performed the abovementioned study selection and data extraction procedures, and another researcher (CYK) cross-checked the process. Any disagreement was resolved through discussion between the researchers.

### 2.4. Quality Assessment

We evaluated the risk of bias for the included RCTs using the Cochrane Collaboration's risk of bias tool [25]. The studies were classified as “low risk,” “unclear,” or “high risk” for each of the following domains: random sequence generation, allocation concealment, blinding of participants and personnel, blinding of outcome assessments, completeness of outcome data, selective reporting, and other potential bias. We evaluated the other potential bias domain with an emphasis on possible baseline imbalances between the treatment and control groups, such as the baseline BP level.

Using the Grading of Recommendations, Assessment, Development and Evaluation (GRADE) approach, we evaluated the quality of evidence for major findings with the online program GRADEpro (https://gradepro.org/) [26]. The risk of bias, inconsistency, indirectness, and imprecision of the results and the probability of publication bias were evaluated, and we classified the results into one of four groups: “very low,” “low,” “moderate,” or “high.”

One researcher (BL) conducted the abovementioned quality assessment, while another researcher (CYK) cross-checked the data. Any discrepancy was resolved through discussion between them.

### 2.5. Data Synthesis and Analysis

We conducted a narrative synthesis of the details of the participants, interventions, comparators, and outcomes for all included studies. In the case of primary or secondary outcome of this review, we quantitatively pooled the results using the Review Manager software, version 5.3 (Cochrane, London, UK). Continuous or dichotomous variables were pooled using mean differences (MD) or risk ratios (RRs), with 95% confidence intervals (CIs). We evaluated heterogeneity between the studies included in each meta-analysis in terms of the effect measures using both the *χ*^2^ test and the *I*^*2*^ statistic. *I*^*2*^ values ≥50% and ≥75% were considered indicative of substantial and considerable heterogeneity. The meta-analyzed results were pooled using a random-effect model if the included studies had significant heterogeneity (an *I*^*2*^ value ≥50%). A fixed-effect model was used when the heterogeneity was not significant or the number of studies included in the meta-analysis was very small, which leads to poor precision for the estimate of between-study variance [27, 28]. Subgroup analysis was conducted according to the type of active controls used. Sensitivity analyses were planned to identify the robustness of meta-analysis results by excluding [1] studies with high risks of bias and [2] outliers. We also planned to assess the evidence of publication bias using a funnel plot if there were enough studies.

## 3. Results

### 3.1. Description of Studies

**Figure 1:**
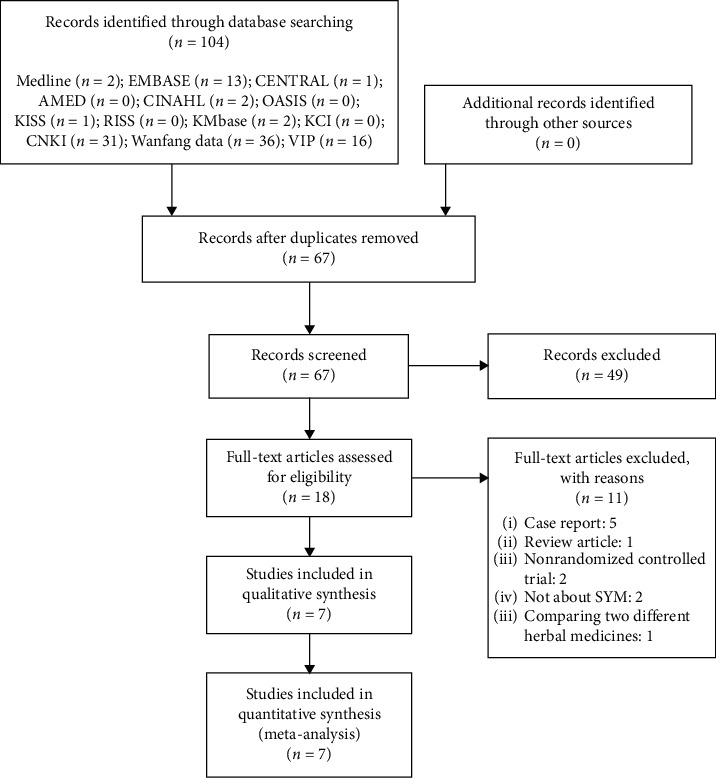
A PRISMA flow diagram of the literature screening and selection process. AMED, Allied and Complementary Medicine Database; CENTRAL, Cochrane Central Register of Controlled Trials; CINAHL, Cumulative Index to Nursing and Allied Health Literature; CNKI, China National Knowledge Infrastructure; KCI, Korea Citation Index; KISS, Korean studies Information Service System; KMbase, Korean Medical Database; OASIS, Oriental Medicine Advanced Searching Integrated System; RISS, Research Information Service System; SYM, Sihogayonggolmoryeo-tang.

A total of 104 articles were identified from searching 13 databases, and no records were identified through other sources. After removing 37 duplicates, 49 articles were excluded based on screening the titles and abstracts. Through the full-text evaluation, 11 articles, specifically five case reports, one review article, two non-RCTs, two non-SYM-related articles, and one article comparing two different HMs, were excluded. Therefore, seven articles [29–35] with 711 participants were included in the qualitative and quantitative synthesis (Figure 1).

### 3.2. Characteristics of Studies

All studies were conducted in China. One article was a thesis [29], and the rest were journal articles. The included studies were as follows: one study comparing SYM with placebo [33], two studies comparing SYM with active controls [30, 35], and three studies comparing SYM plus active controls with active controls only [29, 32, 34]. One study was a three-arm parallel study comparing SYM, active controls, and SYM plus active controls [31]. For the active controls, antihypertensives including captopril, amlodipine besylate, and benazepril were used in four studies [29, 31, 32, 34] and antidepressants including Deanxit (flupentixol and melitracen) were used in two studies [30, 35]. Four studies included participants with hypertension [29, 31, 32, 34], two studies included participants with both hypertension and anxiety [30, 35], and one study included participants with both hypertension and depression [33]. One study targeted participants with isolated nocturnal hypertension [32]. Regarding the criteria for diagnosing hypertension, six studies were based on the Chinese Hypertension Prevention Guidelines [29–33, 35], and one study was based on the 2018 European guidelines for the prevention and treatment of hypertension [33]. The diagnosis criteria were not listed for one study [34]. Participants were recruited for four studies [29, 32, 33, 35] according to specific pattern identification: three [29, 32, 33] were for ascendant hyperactivity of liver yang, and the remaining study [35] was for yin deficiency with yang hyperactivity. Daytime SBP and DBP were evaluated in five studies [28–30, 32, 34], and nighttime SBP and DBP were measured in two studies [28, 31]. As symptoms are related to hypertension, anxiety was assessed in two studies using the Zung self-rating anxiety score (SAS) [30] and Hamilton anxiety rating scale (HAMA) [35]. Depression was assessed in two studies using the Zung self-rating depression scale (SDS) [30] and patient health questionnaire-9 (PHQ-9) [33], respectively. TER calculated based on BP was evaluated in three studies [29, 32, 34], and TER calculated based on the traditional Chinese medicine (TCM) syndrome score was evaluated in four studies [29, 32, 33, 35]. The incidence of adverse events was reported in three studies [29, 33, 35], and quality of life was assessed in one study [29] using Du's hypertension quality of life scale (Table 1). Two articles [30, 33] reported that the studies had been approved by an institutional review board, and three articles [29, 30, 33] reported that the researchers had received consent from the participants.

Regarding the dosage form, a decoction was used in six studies [29–34], and granules were used in the remaining study [35]. At least one of the original components of SYM, *Bupleuri Radix*, *Fossilia Ossis Mastodi*, *Ostreae Testa*, *Scutellariae Radix*, *Pinelliae Tuber*, *Poria Sclerotium*, and *Cinnamomi Ramulus*, was used in all studies (each 100%). Additionally, *Rhei Radix et Rhizoma* was used in six studies (85.7%) [29–31, 33–35], followed by *Ginseng Radix* or *Codonopsis Pilosulae Radix* [29–31, 33, 35] and *Zingiberis Rhizoma Recens* [29, 30, 33–35] in five studies (71.4%) each. The administration duration varied from 2 weeks to 7 months, with 4 weeks being the most (Table 2).

### 3.3. Risk of Bias Assessment

**Figure 2:**
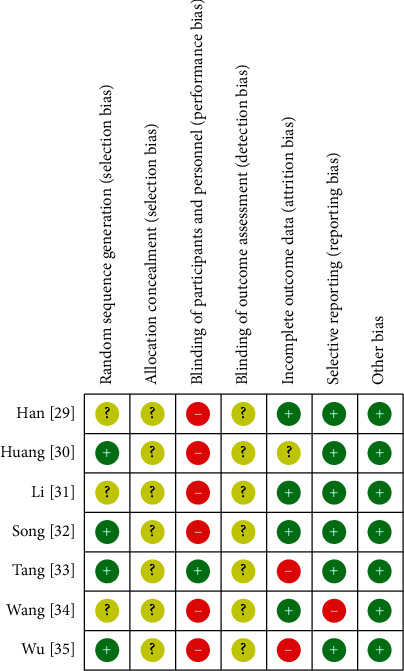
Risk of bias summary for all included studies. Low, unclear, and high risk, respectively, are represented with the following symbols: “+,” “?,” and “−.”

For the random sequence generation domain, four studies [30, 32, 33, 35] were evaluated as having a low risk of bias because they used a random number table, and the remaining three studies [29, 31, 34] were evaluated as having an unclear risk of bias because the relevant information was not provided. None of the articles provided information on allocation concealment and the blinding of outcome assessment; hence, the risk of bias was assessed as unclear. For the blinding of participants and personnel, one study [33] using placebo as a control was judged to have a low risk of bias, and the remaining studies [29–32, 34, 35] without any related information were judged to have a high risk of performance bias given the nature of the interventions used. For the incomplete outcome data domain, one study [30] was determined to have an unclear risk of attrition bias because the related information was not provided, and two studies [33, 35] were judged to have a high risk of bias due to analysis of the results using a per-protocol analysis method. One study [34] that reported only the TER calculated based on BP without reporting the raw data was evaluated to have a high risk of reporting bias. All studies were assessed to have a low risk of bias in other bias domains because they had demographic and clinical homogeneity at the baseline between the treatment and control groups (Figure 2).

### 3.4. Efficacy

#### 3.4.1. SYM versus Placebo

Compared with placebo, SYM resulted in significantly lower daytime SBP and DBP (SBP: one study [33], MD −8.84 mmHg, 95% CI −15.36 to −2.32; and DBP: one study [33], MD −9.11 mmHg, 95% CI −12.03 to −6.19). Additionally, depression measured with PHQ-9 was significantly lower in the SYM group (one study [33], MD −3.52, 95% CI −4.40 to −2.64). TER based on the TCM syndrome score after treatment was significantly higher in the SYM group than in the placebo group (one study [33], RR 1.21, 95% CI 1.01 to 1.45) (Table 3).

#### 3.4.2. SYM versus Active Controls

The SYM group showed significantly lower daytime SBP and DBP compared with the active control group (SBP: three studies [30, 31, 35], MD −2.54 mmHg, 95% CI −4.18 to −0.89, *I*^*2*^ = 83%; and DBP: three studies [30, 31, 35], MD −2.67, 95% CI −3.73 to −1.60, *I*^*2*^ = 56%). However, subgroup analysis according to the type of active controls used to resolve considerable heterogeneity showed that daytime SBP was higher with SYM than with antihypertensives (one study [31]; MD 5.00 mmHg, 95% CI 0.16 to 9.84), and daytime DBP showed no significant difference between the SYM and antihypertensives groups (one study [31]; MD 2.20 mmHg, 95% CI −2.73 to 7.13). The degree of anxiety measured using SAS and HAMA showed no consistent results between the SYM and antidepressants groups (SAS: one study [30], MD −10.58, 95% CI −11.57 to −9.59; and HAMA: one study [35]; MD −0.28, 95% CI −0.95 to 0.39). The degree of depression measured with SDS showed significant results in favor of the SYM group (one study [30], MD −10.94, 95% CI −12.17 to −9.71) (Table 3).

#### 3.4.3. SYM plus Active Controls versus Active Controls Alone

Antihypertensives were used as the active controls in all three studies included in this comparison. SYM plus antihypertensives resulted in significantly lower daytime SBP than antihypertensives alone (two studies [29, 31], MD −3.22 mmHg, 95% CI −6.15 to −0.29, *I*^*2*^ = 0%). However, there were no significant differences between these two groups in daytime DBP, nighttime SBP, and nighttime DBP (daytime DBP: two studies [29, 31], MD −2.46 mmHg, 95% CI −5.09 to 0.18, *I*^*2*^ = 72%; nighttime SBP: two studies [29, 32], MD −1.79 mmHg, 95% CI −3.81 to 0.23, *I*^*2*^ = 0%; and nighttime DBP: two studies [29, 32], MD 0.35 mmHg, 95% CI −1.78 to 2.48, *I*^*2*^ = 0%). The results were significant in favor of the SYM group for TER calculated based on BP (three studies [29, 32, 34], RR 1.22, 95% CI 1.09 to 1.37, *I*^*2*^ = 61%) and the TCM syndrome score (two studies [29, 32], RR 1.28, 95% CI 1.08 to 1.52, *I*^*2*^ = 0%). The quality of life measured based on Du's hypertension quality of life scale was significantly improved in the SYM plus antihypertensives group compared with the antihypertensives-alone group (one study [29], MD −9.85, 95% CI −15.87 to −3.83) (Table 3).

#### 3.4.4. Other Results

In one study [31], serum procollagen III, a biomarker of myocardial fibrosis, was significantly lower in the SYM plus benazepril group compared with the SYM alone or benazepril alone group (all, *P* < 0.05). Tang et al. [33] reported that C-reactive protein was significantly lower in the SYM group than in the placebo group (*P* < 0.05). In addition, they reported that endothelium-dependent vasodilation was significantly improved in the SYM group compared with the placebo group (*P* < 0.05) (Table 1).

### 3.5. Safety

Of the seven studies, only three studies (42.86%) [29, 33, 35] reported adverse events. Tang et al.[33] reported two cases of blushing and two cases of constipation in the SYM group, with one case of ankle edema and four cases of constipation in the placebo group (RR 0.81, 95% CI 0.23–2.88). Han [29] reported no adverse events in the SYM group and one case of dry cough in the SYM plus antihypertensives group (RR 0.33, 95% CI 0.01–7.87). Wu et al. [35] reported no adverse events in the SYM and antidepressants groups (Table 3).

### 3.6. Quality of Evidence

In the comparison of SYM with placebo, the quality of evidence for daytime SBP, daytime DBP, and PHQ-9 was graded as “Moderate.” However, TER calculated based on the TCM syndrome score and adverse events was graded as “Very Low.” In the comparison of SYM with active controls and SYM plus active controls with active controls alone, the quality of evidence was graded as “Very Low” to “Moderate” (Table 3). The main reason for the downgrade was the high risk of bias for the included RCTs and the imprecision of the results due to the small sample size and wide CIs. Additionally, when TER was used, the indirectness of the outcome measure also lowered the quality of evidence.

### 3.7. Publication Bias

Because the number of studies included in each meta-analysis was less than 10, the assessment of publication bias through a funnel plot was not conducted.

## 4. Discussion

The aim of this study was to present evidence regarding the effect and safety of SYM for the treatment of hypertension through a comprehensive and systematic search, and seven studies [29–35] were included in the analysis. According to the study results, SYM significantly lowered BP and depressive symptoms in hypertensive patients compared with placebo. However, only one study [33] with an unclear risk of bias was included in this comparison, and no definite evidence could be obtained. SYM significantly lowered daytime BP compared to active control with considerable heterogeneity. Subgroup analysis according to the type of active controls used to resolve significant statistical and clinical heterogeneity revealed a significant decrease in the *I*^*2*^ value. Interestingly, daytime SBP was significantly higher in the SYM group than in the antihypertensives group, but daytime DBP was not significantly different between the two groups. However, compared to the antidepressants group, daytime SBP and DBP were significantly lower in the SYM group. There was no significant difference in the concurrent anxiety symptoms when comparing SYM and antidepressants, but depression symptoms decreased significantly in the SYM group compared with that in the antidepressants group. When SYM plus antihypertensives was compared with antihypertensives alone, daytime SBP was significantly lower. However, daytime DBP and nighttime SBP showed borderline significance, and nighttime DBP had no significant difference between the two groups. However, TER calculated based on BP and quality of life was significantly higher in the SYM plus antihypertensives group. SYM showed no difference in the incidence of adverse events compared to placebo or active control, and the reported adverse reactions were mild and disappeared spontaneously. Most studies included had an unclear risk of bias, and the quality of evidence for the main findings was generally low.

Although hypertension itself is not a life-threatening problem, it can increase the risk of major medical conditions including cardiovascular and cerebrovascular diseases [36]. In addition, hypertension is reported to be associated with anxiety, depression, and insomnia [37–39], and the prevalence of depression among hypertensive patients is known to be moderate, at about 27% [37]. According to the study results, SYM significantly improved the biomarkers of cardiovascular disease including serum procollagen III and C-reactive protein. In addition, given the increased demand for natural products that can control BP [10], the results of this study suggest the potential of SYM as a treatment option especially for hypertension and concurrent depressive symptoms. Modern pharmacological studies of SYM have shown that this HM can exert antidepressant effects by preventing the collapse of the hypothalamopituitary-adrenal (HPA) axis, including dysfunction in the glucocorticoid negative feedback system [16]. Dysfunction in the HPA axis is associated with negative cardiovascular outcomes, including hypertension, and is speculated to link hypertension and psychological stress [40]. Therefore, SYM can be assumed to have a beneficial effect on both hypertension and depression by restoring stress-related dysfunction in the HPA axis. Interestingly, among the studies included in this review, all studies using pattern identification [29, 32, 34, 35] recruited patients with yang hyperactivity, a concept related to psychological stress [41]. These findings support our hypothesis that the therapeutic effects of SYM may be involved in psychological stress and the HPA axis as a mediator for treating high BP and depression. However, the underlying mechanism of SYM on these conditions needs to be further elucidated.

According to previous studies [14], the pattern identification categories for hypertension are fire syndrome, phlegm-fluid retention syndrome, and deficiency syndrome. Among them, fire syndrome can be classified as a syndrome related to the liver, heart, stomach, and intestine. Based on the included studies using pattern identification and our previous study [42], the treatment principles for SYM were aimed at calming the liver and suppressing liver yang hyperactivity. Therefore, it can be assumed that SYM is used by some researchers under the category of fire syndrome, which mainly includes liver qi or liver yang abnormalities. However, only four of the studies included in this review [29, 32, 34, 35] recruited hypertensive patients with specific patterns, and we could not assess the effects of SYM in hypertensive patients according to specific patterns. Therefore, further study is needed to confirm whether SYM is more effective in hypertensive patients with a certain pattern.

There are some limitations to consider when interpreting the results. First, though we could not assess publication bias because less than 10 studies were included in this review, all included studies were conducted in China, suggesting potential reporting bias. Second, the risk of bias in all included studies was generally unclear; thus, the planned sensitivity analysis to identify the robustness of the meta-analysis results could not be conducted. Third, the incidence of cardiovascular and cerebrovascular events is an important outcome in hypertensive patients; however, none of the included studies conducted follow-up assessment after SYM administration. Nevertheless, some observational studies have reported the potential impact of HM on these outcomes. For example, in a previous cohort study [43], it was reported that HM improved the overall survival rate of hypertensive patients with type 2 diabetes. Long-term clinical trials or large-scale cohort studies are needed to evaluate the effects of SYM on cardiovascular and cerebrovascular events or mortality in hypertensive patients. Finally, although fatal adverse events induced by SYM were not observed in the included studies or in our previous study [42], only three studies (42.86%) [29, 33, 35] reported the incidence of adverse events, and therefore, the safety profile of SYM remains unclear. Especially, although there was no age limit in the inclusion criteria for our study, and none of the included studies targeted children or pregnant women. Children may be even more susceptible to the adverse effects of HM because of their immature metabolic enzyme systems and an inappropriate dose per body weight. HMs also have the potential to cause adverse pregnancy outcomes and affect embryonic and fetal development in pregnant women, similar to conventional medication [44]. In this review, we could not find any evidence for the safety of SYM administration in this population, and further studies on the safety of SYM administration in this vulnerable population are needed. Moreover, due to concerns about potential adverse herb-drug interactions [45], the safety of HM in the treatment of hypertension should still be validated in well-designed clinical trials.

However, to the best of our knowledge, this is the first systematic review that comprehensively evaluated the effectiveness and safety of SYM for treating hypertensive patients. In addition, we significantly limited the statistical heterogeneity through subgroup analysis. Additional high-quality RCTs with placebo control and large sample sizes should be conducted to provide conclusive evidence on SYM for hypertensive patients. In particular, to generalize the results, relevant studies should be conducted in other East Asian countries besides China. In addition, there is a need for further experimental studies on the mechanism of SYM in the treatment of hypertension.

## 5. Conclusions

Current evidence suggests that SYM may have positive effects on hypertension and concurrent depressive symptoms, especially compared with placebo or antidepressants, without serious adverse events. However, due to the unclear risk of bias in the included studies and low quality of evidence for the key findings, additional high-quality and placebo-controlled RCTs are needed to draw a definite conclusion.

## Figures and Tables

**Table 1:** General characteristics of the included studies.

Study ID	Sample size (A) : (B)	Mean age (range) (yr)	Population	(A) treatment group	(B) control group	Treatment period	Outcome	Results reported
Han [29]	60 (30 : 30)	56.4 (43–75)	(i) SBP ≥ 140 mmHg and/or DBP ≥ 90 mmHg (ii) Pattern identification^※^: ascendant hyperactivity of liver yang	SYM + (B)	Captopril 12.5 mg bid + amlodipine besylate 5 mg qd (if there was a severe cough caused by captopril, switch to amlodipine besylate 5 mg qd + furosemide 20 mg qd)	8 wks	(1) Daytime SBP (2) Daytime DBP (3) Nighttime SBP (4) Nighttime DBP (5) TER (BP) (6) TCM syndrome score (7) TER (TCM syndrome score) (8) Du's hypertension quality of life scale	(1) N.S (2) N.S (3) N.S (4) N.S (5) N.S (6) (A) < (B)+ (7) (A) > (B) (*P* value was not presented) (8) (A) < (B)+ (total score, physiological symptoms, somatization symptoms, and obsessive-compulsive conditions), (A) < (B)^*∗*^ (sleep status, anger and vitality, anxiety, hostility, depression, and interpersonal sensitivity)

Huang [30]	176 (88 : 88)	(A) 35.51 ± 3.56 (18–65) (B) 34.89 ± 3.41 (18–65)	(i) Essential hypertension (ii) Anxiety (CCMD)	SYM + amlodipine besylate 5 mg 1T qd	Deanxit (flupentixol and melitracen) 0.5 mg 1T bid + amlodipine besylate 5 mg 1T qd	1 mo	(1) Daytime SBP (2) Daytime DBP (3) SAS (4) SDS	(1) (A) < (B)+ (2) (A) < (B)+ (3) (A) < (B)+ (4) (A) < (B)+

Li [31]	80 (27 : 26 : 27)	(A1) 60.7 (42–78) (A2) 59.7 (39–72) (B) 58.7 (38–74)	(i) Grade 1 or 2 hypertension^#^	(A1) SYM (A2) SYM + (B)	Benazepril 10 mg qd	6 mo	(1) Daytime SBP (2) Daytime DBP (3) Serum PCIII	(1) (A1) > (B)^*∗*^, (A2) < (B)^*∗*^, (A1) > (A2)^*∗*^ (2) N.S (3) (A1) > (A2)^*∗*^, (A2) < (B)^*∗*^

Song [32]	94 (47 : 47)	46.4 ± 9.3 (31–64)	(i) Isolated nocturnal hypertension (ii) Daytime BP < 135/85 mmHg; nighttime BP ≥ 120/70 mmHg (iii) Pattern identification: ascendant hyperactivity of liver yang	SYM + (B)	Amlodipine besylate 2.5 mg	2 wks	(1) Nighttime SBP (2) Nighttime DBP (3) TER (BP) (4) TER (TCM syndrome score)	(1) N.S (2) (A) < (B)^*∗*^ (3) (A) > (B)^*∗*^ (4) (A) > (B)^*∗*^

Tang [33]	121 (60 : 61)	(A) 54.18 ± 9.79 (B) 53.84 ± 10.28	(i) Grade 1 or 2 hypertension (ii) Depression (PHQ-9>4) (iii) Pattern identification: ascendant hyperactivity of liver yang	SYM + amlodipine besylate 5 mg qd	SYM placebo + amlodipine besylate 5 mg qd	4 wks	(1) Daytime SBP (2) Daytime DBP (3) PHQ-9 (4) TER (TCM syndrome score) (5) CRP (mg/L) (6) Inner diameter of brachial artery (mm) (7) Endothelium-dependent flow-mediated dilation (%) (8) Endothelium-independent response to glyceryl trinitrate (%)	(1) (A) < (B)^*∗*^ (2) (A) < (B)^*∗*^ (3) (A) < (B)^*∗*^ (4) (A) > (B)^*∗*^ (5) (A) > (B)^*∗*^ (6) N.S (7) (A) > (B)^*∗*^ (8) (A) > (B)^*∗*^

Wang [34]	100 (50 : 50)	56.21 ± 3.72 (42–61)	(i) Essential hypertension	SYM + (B)	Amlodipine besylate 5 mg qd	NR	(1) TER (BP)	(1) (A) > (B)^*∗*^

Wu [35]	80 (40 : 40)	(A) 48.12 ± 8.25 (B) 50.12 ± 2.13	(i) Grade 1 or 2 hypertension (ii) Anxiety (HAMA > 14, CCMD) (iii) Pattern identification: yin deficiency with yang hyperactivity	SYM + Amlodipine besylate 5 mg qd	Deanxit (flupentixol and melitracen) bid + amlodipine besylate 5 mg qd	4 wks	(1) Daytime SBP (2) Daytime DBP (3) HAMA (4) TER (TCM syndrome score)	(1) (A) < (B)^*∗*^ (2) N.S (3) (A) < (B)^*∗*^ (4) (A) > (B)^*∗*^ (head distension, profuse dreaming, vexing heat in the chest, palms, and soles, and dry mouth and throat), N.S (dizziness, headache, constipation, insomnia, inability to sleep, tinnitus, eye redness, impatient and irritability, and lumbar aching and knee flaccidity)

An approach in some East Asian traditional medicines that enables individual treatment by categorizing the signs and symptoms of patients into a series of syndrome concepts. ^*∗*^ and + mean significant differences between two groups, *P* < 0.05 and *P* < 0.01. N.S means no significant difference between two groups, *P* > 0.05. ^∗^An approach in some East Asian traditional medicines that enables individual treatment by categorizing the signs and symptoms of patients into a series of syndrome concepts. ^#^grade 1: SBP 140–159 mmHg and/or DBP 90–99 mmHg/grade 2: SBP 160–179 mmHg and/or DBP 100–109 mmHg. BP, blood pressure; CCMD, Chinese classification of mental disorders; CRP, C-reactive protein; DBP, diastolic blood pressure; HAMA, Hamilton anxiety rating scale; PCIII, procollagen III; PHQ-9, patient health questionnaire-9; SAS, Zung self-rating anxiety scale; SBP, systolic blood pressure; SDS, Zung self-rating depression scale; SYM, Sihogayonggolmoryeo-tang; TCM, traditional Chinese medicine; TER, total effective rate.

**Table 2:** Details of Sihogayonggolmoryeo-tang in the included studies.

Study ID	Dosage form	Administration duration and frequency	Dosages of components per day (g)																					
			Bupleuri Radix	Fossilia Ossis Mastodi	Ostreae Testa	Scutellariae Radix	Ginseng Radix or Codonopsis Pilosulae Radix	Pinelliae Tuber	Poria Sclerotium	Cinnamomi Ramulus	Zingiberis Rhizoma Recens	Zizyphi Fructus	Rhei Radix et Rhizoma	Minium	Nardotidis seu Sulculii Concha	Margaritifera Usta Concha	Magenetitum	Pseudostellariae Radix	Paeoniae Radix	Glycyrrhizae Radix et Rhizoma	Prunellae Spica	Gastrodiae Rhizoma	Uncariae Ramulus cum Uncus	Puerariae Radix
Han [29]	Decoction	8 wks, bid	15	20	20	9	10	9	15	10	9	6 pieces	6			10								
Huang [30]	Decoction	1 mo, bid	15	20	20	20	10	10	20	5	10	6 pieces	3				30							
Li [31]	Decoction	6 mo, bid	NR	NR	NR	NR	NR	NR	NR	NR			NR	NR	NR									
Song [32]	Decoction	2 wks, bid	10	15	15	10		10	30	10								15	10					
Tang [33]	Decoction	4 wks, bid	15	30	30	10	10	9	30	10	6	15	3							10	15	15	10	30
Wang [34]	Decoction	NR, bid	12	30	30	6		6	18	3	6		6											
Wu [35]	Granule	4 wks, bid	NR	NR	NR	NR	NR	NR	NR	NR	NR		NR											
Frequency (%)			7 (100)	7 (100)	7 (100)	7 (100)	5 (71.4)	7 (100)	7 (100)	7 (100)	5 (71.4)	3 (42.9)	6 (85.7)	1 (14.3)	1 (14.3)	1 (14.3)	1 (14.3)	1 (14.3)	1 (14.3)	1 (14.3)	1 (14.3)	1 (14.3)	1 (14.3)	1 (14.3)

NR, not reported.

**Table 3:** Summary of findings.

Outcomes		No. of Participants (RCTs)	Anticipated absolute effects (95% CI)		Relative effect (95% CI)	*I* ^*2*^ value	Quality of evidence (Grade)	Comments
			Risk with control group	Risk with SYM group				
*SYM versus placebo*								
Daytime SBP (mmHg)	Total	113 (1)	—	MD 8.84 lower (15.36–2.32 lower)	—	Not applicable	⊕⊕⊕⃝ Moderate	Risk of bias (−1)
Daytime DBP (mmHg)	Total	113 (1)	—	MD 9.11 lower (12.03–6.19 lower)	—	Not applicable	⊕⊕⊕⃝ Moderate	Risk of bias (−1)
PHQ-9	Total	113 (1)	—	MD 3.52 lower (4.40–2.64 lower)	—	Not applicable	⊕⊕⊕⃝ Moderate	Risk of bias (−1)
TER (TCM syndrome score)	Total	113 (1)	737 per 1,000	892 per 1,000 (744–1,000)	RR 1.21 (1.01–1.45)	Not applicable	⊕⃝⃝⃝ Very low	Risk of bias (−1) Indirectness (−1) Imprecision (−1)
Adverse event	Total	113 (1)	88 per 1,000	71 per 1,000 (20–253)	RR 0.81 (0.23–2.88)	Not applicable	⊕⃝⃝⃝ Very low	Risk of bias (−1) Imprecision (−2)

*SYM versus active controls*								
Daytime SBP (mmHg)	Total	305 (3)	—	MD 2.54 lower (4.18–0.89 lower)	—	83%	⊕⃝⃝⃝ Very low	Risk of bias (−1) Inconsistency (−2)
	Versus antihypertensives	54 (1)	—	MD 5.00 higher (0.16–9.84 higher)	—	Not applicable	⊕⊕⃝⃝ Low	Risk of bias (−1) Imprecision (−1)
	Versus antidepressants	251 (2)	—	MD 3.52 lower (5.26–1.77 lower)	—	15%	⊕⊕⊕⃝ Moderate	Risk of bias (−1)
Daytime DBP (mmHg)	Total	305 (3)	—	MD 2.67 lower (3.73–1.60 lower)	—	56%	⊕⊕⃝⃝ Low	Risk of bias (−1) Inconsistency (−1)
	Versus antihypertensives	54 (1)	—	MD 2.20 higher (2.73 lower–7.13 higher)	—	Not applicable	⊕⃝⃝⃝ Very low	Risk of bias (−1) Imprecision (−2)
	Versus antidepressants	251 (2)	—	MD 2.90 lower (4.00–1.81 lower)	—	0%	⊕⊕⊕⃝ Moderate	Risk of bias (−1)
SAS	Total (antidepressants)	176 (1)	—	MD 10.58 lower (11.57–9.59 lower)	—	Not applicable	⊕⊕⊕⃝ Moderate	Risk of bias (−1)
SDS	Total (antidepressants)	176 (1)	—	MD 10.94 lower (12.17–9.71 lower)	—	Not applicable	⊕⊕⊕⃝ Moderate	Risk of bias (−1)
HAMA	Total (antidepressants)	75 (1)	—	MD 0.28 lower (0.95 lower–0.39 higher)	—	Not applicable	⊕⃝⃝⃝ Very low	Risk of bias (−1) Imprecision (−2)
Adverse event	Total (antidepressants)	75 (1)	0 per 1,000	0 per 1,000 (0–0)	Not estimable	Not applicable	⊕⊕⃝⃝ Low	Risk of bias (−1) Imprecision (−1)

*SYM plus active controls versus active controls*								
Daytime SBP (mmHg)	Total (antihypertensives)	113 (2)	—	MD 3.22 lower (6.15–0.29 lower)	—	0%	⊕⊕⊕⃝ Moderate	Risk of bias (−1)
Daytime DBP (mmHg)	Total (antihypertensives)	113 (2)	—	MD 2.46 lower (5.09 lower–0.18 higher)	—	72%	⊕⃝⃝⃝ Very low	Risk of bias (−1) Inconsistency (−1) Imprecision (−1)
Nighttime SBP (mmHg)	Total (antihypertensives)	154 (2)	—	MD 1.79 lower (3.81 lower–0.23 higher)	—	0%	⊕⊕⃝⃝ Low	Risk of bias (−1) Imprecision (−1)
Nighttime DBP (mmHg)	Total (antihypertensives)	154 (2)	—	MD 0.35 higher (1.78 lower–2.48 higher)	—	0%	⊕⊕⃝⃝ Low	Risk of bias (−1) Imprecision (−1)
TER (BP)	Total (antihypertensives)	254 (3)	740 per 1,000	903 per 1,000 (807–1,000)	RR 1.22 (1.09 to 1.37)	61%	⊕⃝⃝⃝ Very low	Risk of bias (−1) Indirectness (−1) Imprecision (−1)
TER (TCM syndrome)	Total (antihypertensives)	154 (2)	688 per 1,000	881 per 1,000 (743–1,000)	RR 1.28 (1.08 to 1.52)	0%	⊕⊕⃝⃝ Low	Risk of bias (−1) Indirectness (−1)
Du's hypertension quality of life scale	Total (antihypertensives)	60 (1)	—	MD 9.85 lower (15.87–3.83 lower)	—	Not applicable	⊕⊕⃝⃝ Low	Risk of bias (−1) Imprecision (−1)
Adverse event	Total (antihypertensives)	60 (1)	33 per 1,000	11 per 1,000 (0–262)	RR 0.33 (0.01 to 7.87)	Not applicable	⊕⃝⃝⃝ Very low	Risk of bias (−1) Imprecision (−2)

BP, blood pressure; CI, confidence interval; DBP, diastolic blood pressure; HAMA, Hamilton anxiety rating scale; MD, mean difference; PHQ-9, patient health questionnaire-9; RCT, randomized controlled trial' RR, risk ratio; SAS, Zung self-rating anxiety scale; SBP, systolic blood pressure; SDS, Zung self-rating depression scale; SYM, Sihogayonggolmoryeo-tang; TCM, traditional Chinese medicine; TER, total effective rate.

## Data Availability

The data used to support the findings of this study are included within the article.

## Supplementary Materials

Supplementary Materials Supplement 1. Search terms used in each database. Supplement 2. PRISMA 2009 checklists.
